# Longitudinal study of changing inspirations and aspirations of first-year students before and after the Covid-19 pandemic

**DOI:** 10.1042/ETLS20253025

**Published:** 2026-01-28

**Authors:** Charlotte Boyd, David P. Smith, Melissa M. Lacey

**Affiliations:** 1 School of Biosciences and Chemistry, Sheffield Hallam University, U.K.

**Keywords:** aspiration, careers, curriculum, inspiration, motivation

## Abstract

This longitudinal study investigates the evolving inspirations and aspirations of first-year undergraduate students in Biosciences and Chemistry at Sheffield Hallam University over a five-year period (2018–2023). Students submitted reflective essays during Welcome Week, detailing their motivations for choosing their course and future career goals. Thematic analysis identified six recurring themes: named career, work experience, further study, experience of disease, outreach experience and Covid-19. While most themes remained stable over time, Covid-19 emerged in 2020 and peaked in 2021. Bivariate analysis revealed that Asian/Asian British and Black/Black British students were more likely to cite named careers as an aspiration for study, whilst marginalised ethnic groups were more likely to aspire to further study than their White/White British peers. The findings highlight the importance of aligning curriculum content with students’ career goals and further study aspirations to enhance engagement and motivation. Recommendations include embedding diverse career pathways into course content, supporting progression into postgraduate study, and expanding outreach and work experience opportunities.

## Background

Student inspirations and aspirations for attending university vary and change over time, depending on the current climate and their experiences. New learners’ aspirations reflect their social capital and are informed via the relationship between student and teacher through support and guidance [1,2]. Career aspirations of the individual act as motivation for studying their course, with widening participation agendas aiming to raise and support aspirations [3,4].

Students’ inspirations and aspirations are of interest to higher education institutes as they affect students’ motivation, engagement and attainment. Inclusive curriculum design provides equal opportunity for all students to achieve their programme’s learning outcomes, irrespective of background, demographics, inspirations or aspirations [5,6].

We have previously explored the influence of inspirations and aspirations on student motivation during their first year of study [7], identifying notable variations in career ambitions across different ethnic groups. Aspirations towards careers in medicine and laboratory sciences were significantly different between ethnically marginalised students and their White peers. Insights from focus groups conducted at the end of the first year reveal that students demonstrate heightened motivation and engagement when they perceive a clear alignment between course content and their career goals. In contrast, students report disengagement when the curriculum appears disconnected from their intended professional pathways [7].

Here, 500-word reflective essays written by first-year students were collected over five consecutive cohorts (2018–19 to 2022–23) to determine if and how students' motivations and aspirations changed over time.

## Methods

### Participants

Student participants were first-year cohorts on Biomedical Science, Biochemistry, Biology and Human Biology (grouped as Biosciences) and Chemistry undergraduate degree programmes at Sheffield Hallam University. These Biosciences and Chemistry undergraduate courses enrol ∼250–300 students per year.

### Participant recruitment

First-year students were asked to write up to 500 words on their inspirations, aspirations and achievements at the start of Welcome Week. The task was introduced in a session hosted by their respective course leaders. The students were informed:

“What you write here will be collected by your course leader and given to your academic advisor so we can support you better. This will also form part of your portfolio for one of your modules, you’ll be able to alter what you have written here before you submit your portfolio if you wish, and we’ll tell you more about this piece of coursework in the first few weeks of your course.*”*

The attendance at this session varied between ~60 and 90%. To ensure all students were informed about the study, students were also notified about the task by email at the end of Welcome Week. To incentivise students to complete the task, the written reflection formed the introduction page of their skills portfolio, which is their coursework for their first year skills module. This introduction did not bear any weighting in the coursework, and students were informed that submitting their reflection in the study for analysis was completely voluntary and would not affect their grade.

Participants submitted their reflective written task to a Google Form, which also asked them about their course, gender and ethnicity, as well as if they consented to be part of the study. Students were also prompted to save a copy of their reflection to use in their portfolio introduction.

### Reflections written task

The reflective task instructions were:

“We’d like to know a little bit about you before your course starts, so we’d like you to write about 500 words on your achievements, inspirations and aspirations. Please write about any achievements linked to your course, what inspired you to apply to your course and what your aspirations are for when you have completed your course. Some students come onto their course knowing exactly what job they want upon graduation whereas others have no idea. If you fall into the latter category, please write about the type of jobs you think you’d like, would you like to work in a laboratory? or would you like to work in a customer facing role? for example.”

### Ethics

Ethics for this study was acquired through the Faculty of Health and Wellbeing ethics committee following the Sheffield Hallam University Research Ethics Policy (reference: ER8927046). Students were asked to write 500 words about their inspirations and aspirations in Welcome Week and gave consent to be part of the study. Student names were removed from the submissions before analysis.

### Data analysis

A thematic analysis was undertaken of written reflections across the five years of the study [8]. Due to the large amount of data to be analysed, an inductive/deductive hybrid thematic analysis approach was used [9]. Briefly, a sample (*n*=10) from each cohort was open-coded by two independent researchers, and initial themes were identified. The two researchers then discussed the identified themes and compared them to the previous study [7]. To ensure coding saturation, another sample (*n*=5) from each cohort was open-coded using the study themes, identified as named career, work experience, further study, experience of disease, outreach experience and Covid-19 (Table 1).

**Table 1:** Coding strategy for each theme

Theme	Coding notes
Named career	Student specified a career they want after graduating within their essay.
Work experience	Mention of online or in-person work experience within their essay. Mentioning a job close to their degree, such as a carer, was also classed as work experience. Summer schools were classified as work experience, while online courses were not.
Further study	Further study includes a Postgraduate Certificate in Education (PGCE), a master’s or a doctorate. Where students noted a career where further study is required, e.g. postgraduate medicine or physician associate, further study was recorded.
Experience of disease	Students had any personal or familial experience with disease or illness.
Outreach experience	Outreach experience or activity by a university or other group.
Covid-19	This included any mention of the terms ‘Covid-19’, ‘Covid’, ‘pandemic’, ‘lockdown’ or ‘coronavirus’ within the essay.

Using this coding strategy, the full data set was closed-coded by one researcher. Briefly, each transcript was given a unique code, and the presence or absence of each theme was determined. These data were collated in Excel and combined with the student’s course, gender and ethnicity reported by the participant when they submitted their reflection to the study. Bivariate analysis was undertaken to determine if student gender, ethnicity or course of study impacted the themes.

## Results

### Students’ inspirations and aspirations to undertake their degree

This study set out to investigate Bioscience and Chemistry undergraduate students’ inspirations and aspirations for studying their chosen course over five years. Participation in the study varied by cohort, with between 40% and 75% opt-in rate. 2018–19 had 202 participants of a cohort of 268 (75% opt-in rate), 2019–20 had 107 participants of a cohort of 273 (40% opt-in rate), 2020–21 had 150 participants of a cohort of 277 (54% opt-in rate), 2021–22 had 113 participants of a cohort of 240 (47% opt-in rate) and 2022–23 had 155 participants of a cohort of 213 (73% opt-in rate).

Six distinct themes were identified within the participants’ reflections of their inspirations and aspirations to undertake their degree (Table 2).

**Table 2:** Inspirations and aspiration themes

Theme	Example text and year written
Named career	“...I would hope to go into cancer research in the future.” – 2018
Work experience	“...undertake a virtual work experience at the [institute] medical school” – 2022
Further study	“...study the Physician associate masters…” – 2019
Experience of disease	“I was diagnosed with congenital heart disease.” – 2021
Outreach experience	“Being a part of the Discover STEM course at [institute]” – 2020
Covid-19	“During the pandemic and leading up to coming to university, I regularly helped at COVID vaccination clinics” – 2022

### Students’ inspirations and aspirations did not change across the five cohorts

To determine if the frequency of named themes altered over the course of the five-year study, students’ written reflections were coded, and the frequency of each theme within each year’s cohort was determined (Table 3). Unsurprisingly, the theme Covid-19 first emerged in 2020, with 13% of students mentioning the pandemic in 2020, with this more than doubling to 28% in 2021 (*P*<0.02). The number of students who indicated the themes over the longitudinal study was stable for named career, work experience, further study, disease and outreach, with no significant difference observed over the course of the study. This result would demonstrate that students’ aspirations and reasons for choosing to study are relatively constant and hence are suitable as a basis for long-term strategic curriculum planning.

**Table 3:** Longitudinal inspirations and aspirations. Percentages are the number of student reflections in which a theme occurs within a cohort. Percentages of themes do not equal 100%, as students’ reflections may encompass more than one theme. *n* values given are the number of participants’ reflections analysed per cohort. * Indicates *P*<0.05 in a Chi-square test

Theme	2018–**19** *n*=202	2019–**20** *n*=107	2020–**21** *n*=150	2021–**22** *n*=113	2022–**23** *n*=155
Named career	69 (34%)	29 (27%)	45 (30%)	35 (30%)	32 (20%)
Work experience	71 (35%)	52 (49%)	54 (36%)	33 (29%)	59 (38%)
Further study	36 (18%)	26 (24%)	43 (29%)	26 (23%)	39 (25%)
Experience of disease	23 (11%)	16 (15%)	19 (13%)	16 (14%)	32 (21%)
Outreach experience	26 (13%)	19 (18%)	18 (12%)	8 (7%)	20 (13%)
Covid-19*	-	-	20 (13%)	32 (28%)	42 (27%)

### Impact of ethnicity on students’ inspirations and aspirations

Within the context of UK Higher Education, the Ethnicity Degree Awarding Gap remains present, despite well over a decade of interventions since the National Union of Students Race Equality Report [10]. In our previous work, we identified that students from marginalised ethnic groups were more likely to aspire to medical or dentistry careers, whilst White students were more likely to aspire to laboratory work-based careers [7]. DeWitt et al. identified differences within marginalised ethnic groups in terms of career aspirations, with students from Asian/Asian British backgrounds being more likely to have high career aspirations (e.g. medicine, dentistry) than their White/White British peers [11]. To determine if students’ ethnic backgrounds influenced their inspirations and aspirations in this longitudinal study, a bivariate analysis was undertaken to compare ethnicity and named theme (Table 4).

**Table 4:** Aspiration themes by ethnicity. Percentages are the number of student reflections in which a theme occurs within a named ethnically marginalised group. Percentages do not equal 100%, as reflections encompass more than one theme. * Indicates *P*<0.05 in a Chi-square test. ° Indicates *P*<0.05 in a Chi-square test when marginalised ethnic groups are combined and compared with White/White British students

Theme	Asian/Asian British *n*=118	Black/Black British *n*=40	Other/Mixed *n*=47	White/White British *n*=516
Named career*	47 (40%)	18 (45%)	13 (28%)	130 (25%)
Work experience	47 (40%)	11 (28%)	18 (38%)	191 (37%)
Further study°	32 (27%)	13 (33%)	17 (36%)	108 (21%)
Experience of disease	8 (7%)	6 (15%)	7 (15%)	84 (16%)
Outreach experience	15 (13%)	1 (3%)	3 (6%)	72 (14%)
Covid-19	17 (14%)	7 (18%)	2 (4%)	67 (13%)

Students from Asian/Asian British and Black/Black British demographic backgrounds were statistically more likely to aspire to a named career than students from White/White British backgrounds, for example, medicine, research or dentistry. No difference was seen between ethnic groups and the themes of work experience, experience of disease, outreach experience and Covid-19. No difference was seen based on gender (data not shown). Students from marginalised ethnic groups were also statistically more likely to aspire to further study than students from White/White British backgrounds. However, this is not reflected in the numbers who then move on to further study [10].

## Conclusions

This five-year study examined Bioscience and Chemistry undergraduates’ inspirations and aspirations, identifying six recurring themes: named career, work experience, further study, experience of disease, outreach and Covid-19. All themes remained stable over time except Covid-19, which was first identified in the 2020–21 cohort and peaked in the 2021–22 cohort.

Ethnicity influenced aspirations, with Asian/Asian British and Black/Black British students most likely to cite named careers as an aspiration. In addition, marginalised ethnic groups were more likely to aspire to further study than their White/White British counterparts. No significant differences were found across gender or in themes such as work experience, disease, outreach or Covid-19 across ethnic groups.

Findings here support and expand on our previous work [7] and can be utilised to create an engaging and inclusive curriculum as set out below [5,6,12], especially for those from marginalised ethnic groups. Students’ inspirations and aspirations for choosing to study are relatively constant across this longitudinal study and hence are suitable as a basis for long-term strategic curriculum planning. Taken together, practical recommendations are:

1. Students from Asian/Asian British and Black/Black British backgrounds are more likely to aspire to a named career, for example, medicine, research or dentistry. By ensuring students see careers represented within their course, students will see their aspirations reflected in their studies. As we have previously shown, students from marginalised ethnic groups are likely to have proportionally different career aspirations than their White peers; a range of careers being showcased is vital for inclusivity.
2. Here we have shown that students from marginalised ethnic groups are more likely than their White peers to aspire to further study. To support these aspirations within the curriculum, we recommend linking topics and skills to postgraduate courses and providing support for successful progression into further study.
3. Students have been inspired to study by undertaking work experience before starting their degrees. To maintain this inspiration, we recommend providing a range of work experience and field trips to inspire students and help them gain the skill set that they need [13].
4. Students are inspired by their experience of disease. By providing teaching that is relevant to the students, including their experience of disease, interest within the curriculum can be maintained. This should, however, be handled sensitively, as talk of disease states can be a trigger to some students due to past experiences. In addition, care should be taken not to single out any individual group by overly concentrating on a given disease. A nuanced approach allows students to see themselves and their experiences reflected in their curriculum in a respectful and inclusive manner.
5. The value of outreach in encouraging students to study science at university level is highlighted within this work. These outreach events are vital for first-generation students who have no ‘insider’ knowledge of what university is like from other family members or friends [14–16].

## Data Availability

Data is available upon request.

## Open Access

This article has been published open access under our Subscribe to Open programme, made possible through the support of our subscribing institutions, learn more here: https://portlandpress.com/pages/open_access_options_and_prices#conditional
